# Computational-Assisted Development of Molecularly Imprinted Polymers for Synthetic Cannabinoid Recognition

**DOI:** 10.1021/acsomega.5c03148

**Published:** 2025-07-24

**Authors:** Leonardo Martins Carneiro, Karen Rafaela Gonçalves Araújo, Diego Ulysses Melo, Fernando Heering Bartoloni, Alexandre Learth Soares, Mauricio Yonamine, Paula Homem-de-Mello

**Affiliations:** † Centro de Ciências Naturais e Humanas, 74362 Universidade Federal do ABC, Santo André, São Paulo 09210-580, Brazil; ‡ Department of Clinical and Toxicological Analyses, School of Pharmaceutical Sciences, University of São Paulo, Butantã, São Paulo 05508-000, Brazil; § Department of Organic Chemistry, Institute of Chemistry, 28110 Universidade Federal Fluminense, Outeiro São João Batista, Niterói, Rio de Janeiro 24220-900, Brazil; ∥ Superintendence of the Technical-Scientific Police, Institute of Criminalistics, São Paulo, São Paulo 05507-060, Brazil

## Abstract

Synthetic cannabinoids (SCs), a prominent class of new psychoactive substances, pose growing challenges to public health due to their severe toxic effects and widespread global presence. In this study, we employed computational methods to develop molecularly imprinted polymers (MIPs) for the selective recognition of seven SCs, chosen based on seizure reports from the Narcotics Examination Unit of the Scientific Police of the State of São Paulo. Density functional theory and extended tight binding for geometry, frequency, and noncovalent model 2 (GFN2-xTB) calculations were used to optimize the molecular geometries and predict ideal monomer–solvent combinations for MIP synthesis. We assessed six solvents—acetone, acetonitrile, dichloromethane, chloroform, diethyl ether, and dimethyl sulfoxide—based on their solvation energy, identifying suitable candidates for the polymerization step. Hydrogen bonding interaction sites were mapped, guiding the selection of functional monomers such as acrylic acid (AA), 4-vinylbenzoic acid (BA), 2-(trifluoromethyl)­acrylic acid (TFAA), and methacrylic acid. Our findings suggest that TFAA and BA offer the most stable complexation with SCs, influenced by their acidity and aromatic interactions. These computational predictions pave the way for resource-efficient experimental validation and enhance the development of MIPs as tools for the extraction of SCs in complex matrices, contributing to efforts to combat the global SC epidemic.

## Graphical abstract

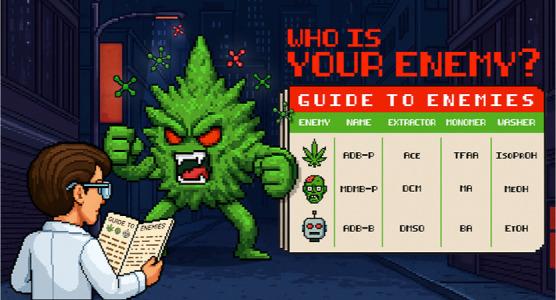

## 1 Introduction

Since the early 2000s, synthetic cannabinoid (SC) receptor agonists, or more simply SCs, have been present in the global scene of new psychoactive substances, posing an increasingly significant challenge for security and public health agencies worldwide.^1^ SCs constitute the largest class of new psychoactive substances, with 353 substances already notified to the United Nations Office of Drugs and Crime (UNODC) by 84 countries through the Early Warning Advisory system.

Mirroring the effects of THC (i.e., (−)*trans*-Δ^9^-tetrahydrocannabinol) on the endocannabinoid system’s CB1 and CB2 receptors, many SCs available on the illicit market today appeared from research aimed at the potential use of THC analogs or substances capable of activating cannabinoid receptors for therapeutic use, such as pain reduction, antiepileptic effect, anti-inflammatory, antiemetic, and antineoplastic activity, without causing dependence.^2,3^ However, many adverse effects are associated with SCs, such as hallucinations, anxiety, suicidal thoughts, rhabdomyolysis, cardiovascular effects, as well as severe intoxications leading to cerebral and cardiac ischemia, heart attack, and even death.^3^

In Brazil, in 2023, the number of intoxication cases involving mixtures of more than one SC increased significantly. According to data from the Municipality of São Paulo, in the first report compiled by the Surveillance Coordination of the Municipal Health Secretariat, there were 493 notifications of suspected cases of SC intoxication in 2023.^4^ In the neighboring state of Rio de Janeiro, SCs have also been identified within prisons, showing similar patterns of substances found in São Paulo, with a greater emphasis on MDMB-4en-Pinaca, ADB-Butinaca, and 5F-MDMB-Binaca.^5^

Given the risk to public health and the imminent global epidemic related to SCs, it becomes imperative to develop methods that assist in the detection of these compounds. One strategy described in the literature is the use of molecularly imprinted polymers (MIPs), which have the ability to selectively extract a specific molecule from a complex matrix.^6^ This is a significant advantage, as these SCs are often found in matrices such as cellulose or vegetables, mixed with other controlled or uncontrolled substances.^7^

MIPs are based on the formation of specific cavities on the surface of a polymer, which mimic the shape of a template molecule.^8^ The application process begins with the interaction of monomers with the target molecule, followed by polymerization of the monomers. Finally, the target molecules are removed, leaving only the desired cavity. In developing an MIP system for a specific molecule, it is essential to determine the optimal combination of monomers and solvents for each stage of the production process.^9,10^

For example, different strategies are being developed to propose MIPs for recognizing certain natural and SCs. These strategies are based on the ability of MIPs to extract specific molecules, followed by trace level detection analysis. In the literature, the most commonly used technique for this detection is liquid chromatography tandem mass spectrometry (LC–MS/MS).^11–14^ However, there are also studies that employ Raman spectroscopy in solution^15^ and voltammetry,^16^ demonstrating the versatility of MIP applications for cannabinoid preconcentration. In addition, Yang et al., in 2023, conducted a study on cannabidiol extraction using MIP-coated magnetic nanoparticles, aiming to improve extraction yield to meet the market demand for therapeutic cannabidiol.^17^

The experimental proposition of MIPs can be resource-intensive, involving significant amounts of reagents and solvents. An alternative to mitigate these costs is the application of computational methods to predict a better combination of monomers and solvents for MIP production.^18,19^ Different computational strategies can be employed to evaluate the steps to produce efficient MIPs, as well described by Mizaikoff et al.^20^ In general, when the template is a macromolecule, as proteins, and to study the polymerization, which involves a large number of atoms, molecular dynamics simulations are the most appropriate strategy.^21^ On the other hand, quantum mechanics-based methods are computationally expensive but accurate in calculating the interactions among the molecules and the possibility of (de)­protonation, for example. Density functional theory (DFT) is a quantum mechanics method with a good cost-accuracy ratio. So, for studying systems with a large number of atoms, DFT methods became very useful, particularly during the prepolymerization steps, since the interactions between the template and the functional monomers (FMs) are key to this stage.^20^

In a previous study,^22^ our group employed a computational approach for the development of MIPs targeting Δ^9^-THC and THC–COOH. To broaden the selection of FMs, the complexation energy between these target molecules and seven different FMs was estimated by using DFT calculations. Furthermore, the effect of four different solvents was incorporated through an implicit solvent model.^22^ This approach is adopted to identify the most suitable FM for a given target molecule and has been successfully applied to other compounds, such as thiamethoxam,^23^ atenolol,^24^ and carvedilol.^25^

Computational methods play a crucial role in these studies by enabling the evaluation of various monomers and solvents and optimizing conditions for subsequent experimental assays. However, these often focus on a single target molecule rather than addressing a broader family of structurally related compounds, such as natural cannabinoids, cathinones, or SCs, where slight structural modifications can lead to significant differences in properties. Furthermore, the implicit solvent model has limitations, especially when used for protic solvents, as it may not accurately capture key solvent–molecule interactions.

So, while MIPs hold immense promise as a cost-effective recognition method, their experimental development often demands significant resources due to the myriad variables at play. Our computational approach contributes to modifying this landscape by assessing polymer composition, solvent selection, and monomers p*K*_a_. The last is an often overlooked but critical factor in such studies, since p*K*_a_ evaluation may ensure that the hydrogen bonds formed in the complexes would be consistent with experimental data. We went beyond the one-molecule-at-a-time approach prevalent in this field,^20^ by applying a combination of the xTB and DFT methods, which allowed for studying systems with a higher number of atoms at a lower computational cost than methods that rely solely on DFT. This approach enabled the addition of underexplored aspects, such as the combination of 7 SCs (templates) with 4 FMs and 4 explicit solvents, resulting in 56 complexes that were studied. In this way, we systematically investigated the optimal conditions to produce MIPs for seven different recently seized SCs reported by the Narcotics Examination Unit of the Scientific Police of the State of São Paulo (NEE-SP).

## 2 Methodology

The selection of the seven SCs studied in this work was carried out in partnership with the Narcotics Examination Unit of the Scientific Police of the State of São Paulo (NEE-SP). The NEE-SP conducted a reference study in 2021 on SCs that were submitted to the institution in cellulose matrices,^26^ and from this report, the three most seized SCs were selected, 5F-MDMB-Pica (**1**), 4F-MDMB-Butinaca (**2**), and MDMB-4en-Pinaca (**3**, Figure 1). The remaining four SCs were selected based on the total number of seizures in the period from July 2022 to December 2023, considering all matrices: MDMB-Butinaca (**4**), ADB-Butinaca (**5**), ADB-4en-Pinaca (**6**), and ADB-Fubinaca (**7**, Figure 1).

**Figure 1:**
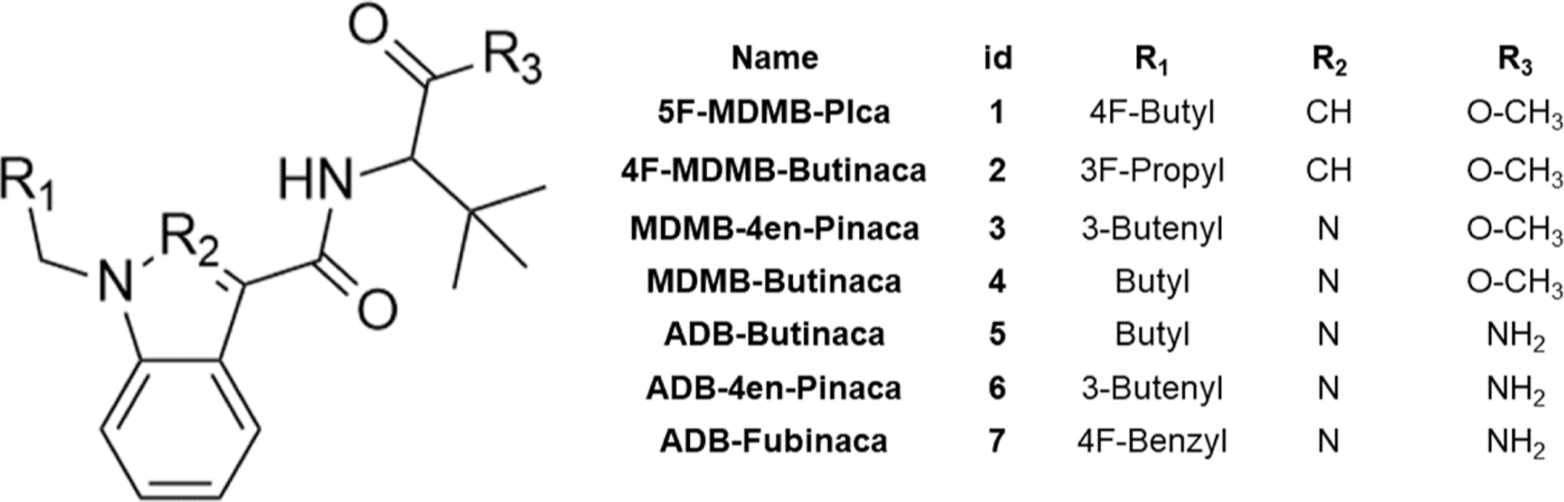
General structural framework of the seven SCs studied in this work, with their particular substituents and functional groups.

Geometry optimization calculations were performed using GFN2-xTB (version 6.5.0)^27^ at the most stringent convergence level, without applying solvent models. Only geometries without imaginary frequencies were considered, indicating minimum energy structures. Total energy calculations were carried out using DFT with the ωB97XD/6–31+G­(d,p) level of theory,^28,29^ as implemented in the Gaussian 09 package.^30^

The study of the interactions is a crucial aspect that requires a thorough evaluation. Initially, SCs are extracted from the matrices where they were commercialized, a process that typically involves the use of aprotic solvents. So, in this case, a continuous solvation model can be employed; here, the solvent effects were incorporated through the solvent model density (SMD) within the DFT framework.^31^ The next step consists of evaluating the more adequate monomer to be used. Atomic charges and the electrostatic surface potential (ESP) were computed for the SCs using the ChelpG scheme,^32^ allowing us to determine the regions for specific interactions with four different monomers. Molecular surfaces and intermolecular interactions were visualized with Binana 2.2 and Jmol 2.1.^33^ The last step consists of washing the MIPs to remove the template (SC). In this case, strong hydrogen bonding may be established among the washing solvent and SCs, so four protic solvents were evaluated for each template. As hydrogen bonding must be evaluated, explicit solvent molecules were included at each proton donor or acceptor group in the calculations.

For the theoretical determination of the p*K*_a_ value, all geometry optimizations were performed at the theoretical level ωB97XD/aug-cc-TZVP, using the implicit solvation model SMD, along with the addition of two explicit water molecules.^34,35^ For more details on this specific determination, see Supporting Information.

## 3 Results and Discussion

### 3.1 Selection of Synthetic Cannabinoids

The SCs studied in this work (Figure 1) have a standard scaffold, which can be divided into four molecular regions: tail, core, linker, and linked group (Figure 2).^1^ For each of these regions, there is a wide variety of modifications; thus, the total number of synthetically possible cannabinoids is significantly large. However, there is a predominance, not only in Brazil but also worldwide, of a small recurrent number of derivatives. In other words, the SCs with the highest number of seizures also appear among the most prevalent SCs worldwide.^36^

**Figure 2:**
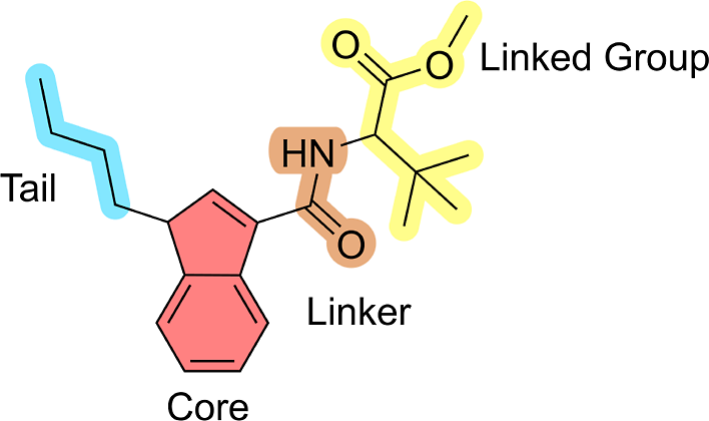
Scaffold of SCs with four molecular regions highlighting the tail (in blue), core (in red), linker (in orange), and linked group (in yellow).

Therefore, the various substitutions recurrently found in SCs and that were studied in this work (Figure 1) within the context of MIP production are, specifically, tail substitutions (R_1_, including butyl, pent-4-enyl, 4-fluorobenzyl, 4-fluorobutanyl, and 5-fluoropentanyl groups) and two core structures, indole (R_2_ = CH) and indazole (R_2_ = N). The linker utilized in our study was carboxamide (i.e., CONH_2_), and the groups attached to this linker were *tert*-leucinamide and its methyl ester.

### 3.2 Solvent Selection for the Extraction of SC from the Matrices

Strategically, to promote the association between a given SC and the monomer of an MIP through hydrogen bonding interactions, the polymerization process should occur in an organic, aprotic, and polar solvents. This allows for the solvation of the reagents of interest without competition with hydrogen bonding interactions that could arise from the solvent. Six solvents were selected for this purpose: acetone (Ace), acetonitrile (ACN), chloroform (Chl), dichloromethane (DCM), diethyl ether (EtOEt), and dimethyl sulfoxide (DMSO). To obtain the total solvation energy of the molecule (*E*_sol(SMD)_), the difference between the total energies of the molecule in vacuum (*E*_SC_) and in the presence of solvent (*E*_SMD_) was determined, following the SMD formalism, according to eq 1.

1 $$E_{sol\left(SMD\right)}=E_{SC}-E_{SMD}$$

In general, from our data, Ace, ACN, and DCM emerge as the best candidates for the polymerization stage as well as for the extraction of the SC from the seizure matrices, given the higher interaction (i.e., more negative *E*_sol(SMD)_) with the SCs. Thus, we can conclude that solvents with moderate dipole moment values are preferable. Both the solvents that are the most and least polar, respectively, DMSO and EtOEt, did not present satisfactory solvation properties in the context of the analysis above, considering their lower interaction (i.e., less negative *E*_sol(SMD)_) values (Figure 3—all values are also presented in Table S3).

**Figure 3:**
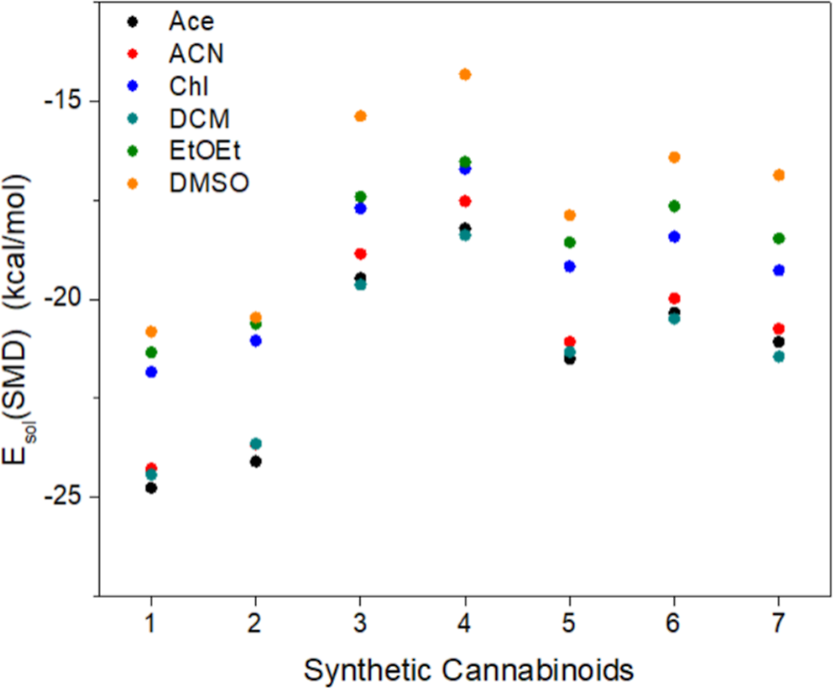
Total solvation energy for the SCs in the solvents acetone (Ace), acetonitrile (ACN), chloroform (Chl), dichloromethane (DCM), diethyl ether (EtOEt), and dimethyl sulfoxide (DMSO), calculated with the SMD model.

### 3.3 Selection of Reaction Sites

The cannabinoid molecules were optimized using the semiempirical GFN2-xTB method, followed by determination of total energy in vacuum, with the theoretical level ωB97XD/6–31+G­(d,p). ChelpG maps (within a range of −0.1 to 0.1 eÅ^–3^) were plotted to better identify the reaction sites (Figure 4). In the Supporting Information, we provide all the representations of the electrostatic potential surfaces presented with the 2D molecular structures (Table S4).

**Figure 4:**
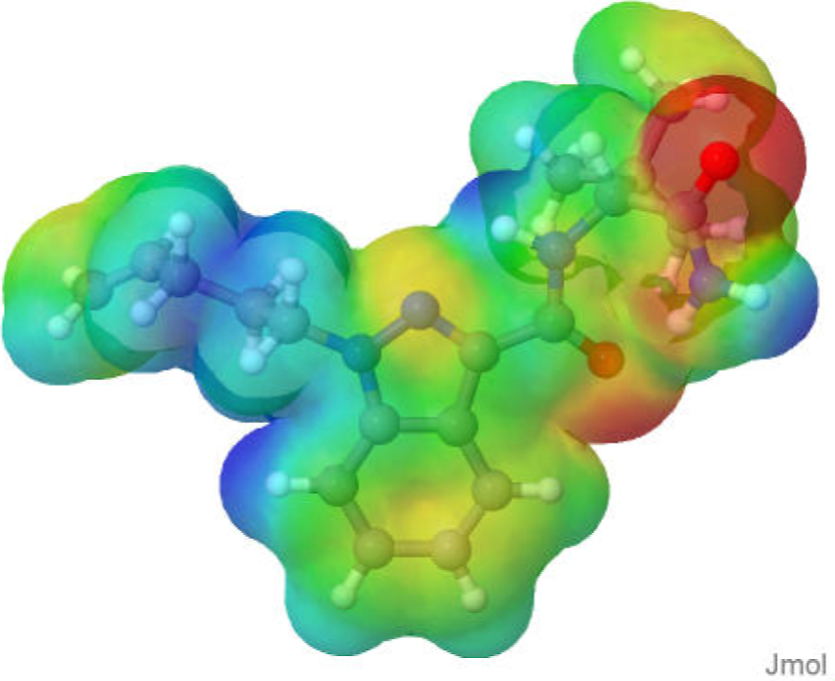
ESP for molecule **6**, where negative density regions are represented by red color, positive by blue color, and intermediate by green color.

Cannabinoids **1**, **2**, and **7** have three regions with a negative charge density, while the other compounds have only two regions with a negative charge density. After the regions of intramolecular interaction were disclosed, three patterns of HB formation were identified. The first pattern occurs only in SCs with a fluorine atom at the end of the tail group. The second pattern occurs in SCs of the MDMB class, which have an amide group and an ester group, with an arrangement that allows interaction with a HB donor group and another HB acceptor group in one face of the molecule and interaction with a HB donor group in the other face of the molecule, as shown in Figure 5a; such MDMB SCs are molecules **1**, **2**, **3**, and **4**. The third pattern occurs in the ADB class, which is composed of two amide groups; thus, on both faces of the molecule, there will be the possibility of interaction with an HB donor group and another HB acceptor group on each side, as shown in Figure 5b. All the aforementioned interaction patterns are suitable for carboxylic acid monomers, as this functional group can participate in hydrogen bonding both as a donor and an acceptor.

**Figure 5:**
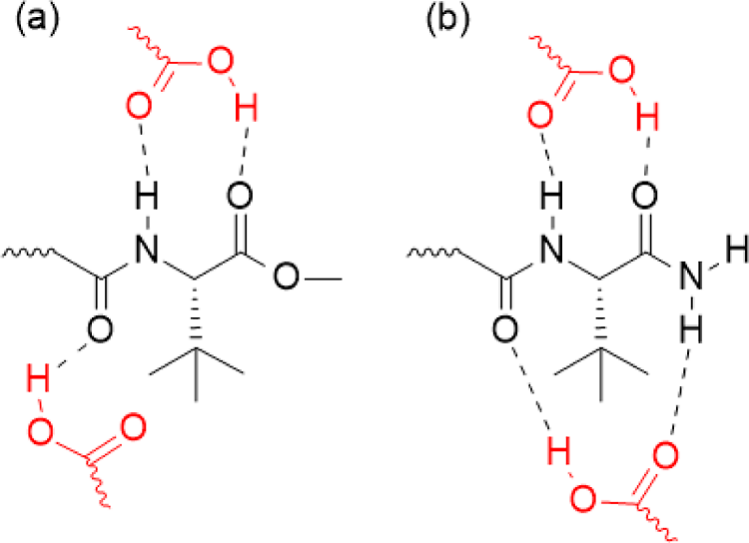
Pattern for HB formation between a carboxylic acid and an MDMB group (a) and ADB group (b).

### 3.4 Selection of the Functional Monomer

With the identification of potential intramolecular interaction points of HB nature and the selection of the carboxylic acid functional group as the most suitable within this context, among the selected FMs, there are acrylic acid (AA), methacrylic acid (MA), 2-(trifluoromethyl)­acrylic acid (TFAA), and 4-vinylbenzoic acid (BA), Figure 6. After the positioning of the monomers, considering the interactions and their quantities relative to each SC as described above, geometry optimization was performed by using the GFN2-xTB method. Upon obtaining the geometry at a minimum in the potential energy surface, the total energy was calculated using ωB97XD/6–31+G­(d,p).

**Figure 6:**
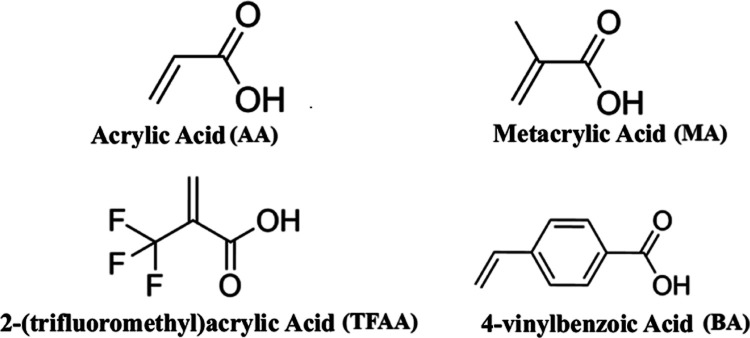
FMs applied in this work, with their respective names and abbreviations.

The total complexation energy (*E*_complexation_) was determined using the total energies of the generated complex (*E*_SC–FM_), the isolated SC in vacuum (*E*_SC_), the isolated FM in vacuum (*E*_FM_), and the number of monomers present in each complex (*n*), according to eq 2.

2 $$E_{complexation}=E_{SC-FM}-\left[\left(n\times E_{FM}\right)+E_{SC}\right]$$

Among the evaluated FMs, TFAA and BA exhibited the strongest interaction, as indicated by the *E*_complexation_ values, meaning they were the FMs that most stabilize the SCs, as shown in Figure 7 (and Table S5). This behavior parallels the trend observed in the p*K*_a_ values of these acids, which are organized in increasing order, as follows: TFAA, AA, BA, and MA (see Table S3 for theoretical and experimental p*K*_a_ values).^34,35,37,38^ The acidity of the monomers decreases in this order; thus, the ability to donate a hydrogen-bonding to a Lewis base (i.e., the SC framework) can be seen as increasing; indeed, TFAA presents the highest complexation energy (Figure 7) and lowest p*K*_a_ value among the evaluated FMs.

**Figure 7:**
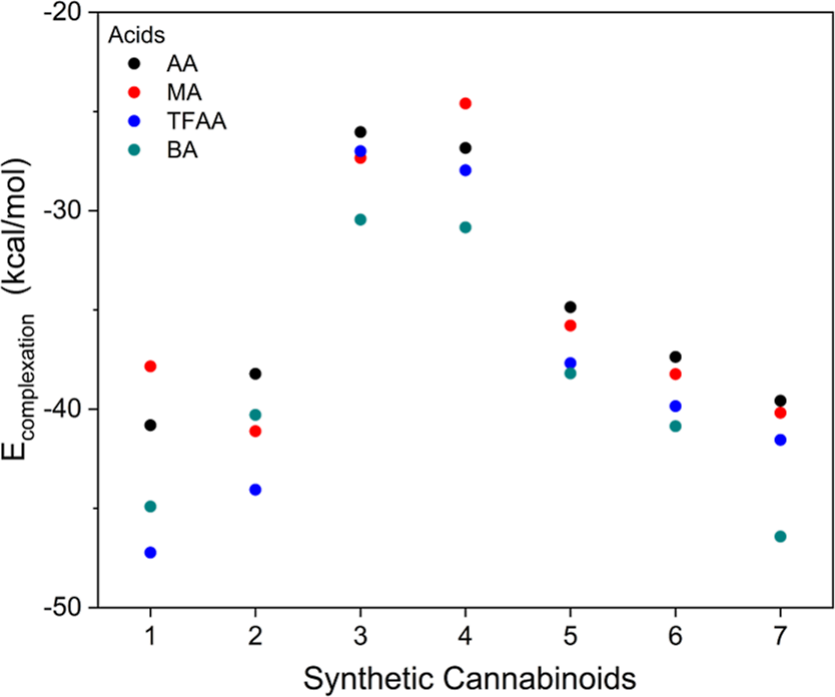
Complexation energy (*E*_complexation_) for SCs and FMs.

Despite BA having a p*K*_a_ value higher than that of TFAA, BA possesses an additional π-stacking interaction. In most complexes formed with BA, its aromatic ring is positioned parallel to the aromatic ring of the SC’s core, especially in the case of **7**, which features an aromatic ring in its apolar extension, favoring this additional interaction.

Observing the range of complexation energies for each SC with a given FM (Figure 7), we can divide the SC into four groups. SCs with a fluorine atom in the tail moiety and MDMB as the linked group: **1** and **2**; SCs formed with a tail moiety consisting only of an aliphatic chain and MDMB as the linked group: **3** and **4**; SCs without the fluorine atom in the tail moiety and ADB as the linked group: **5** and **6**; and a last group consisting only of the SC **7**, with an aromatic ring and a fluorine atom in the tail moiety, and the presence of the ADB group as the linked group.

In Figure 8, all interactions between a representative of the four groups mentioned above and the FM resulting in the strongest interaction complexation energy are observed, and the other SCs and their interactions are presented in Table S6. For **2** and **6** (Figure 8a,c), four possible hydrogen bonds (black arrows, Figure 8) are formed between the ADB and the carboxylic acid groups, in addition to the hydrophobic interaction (gray circles, Figure 8) between the tail moiety and the aromatic ring present in the BA monomer. Justifying this set as being the strongest interaction, **2** was the only SC that exhibited a stronger interaction with FM TFAA (Figure 8a). Despite TFAA performing a hydrophobic interaction of lesser intensity compared to BA, it has a greater hydrogen bonding capacity than BA, and thus, the existence of a HB with the fluorine atom present in the tail moiety provides a higher binding energy with TFAA of −44.1 kcal/mol, as shown in Figure 7.

**Figure 8:**
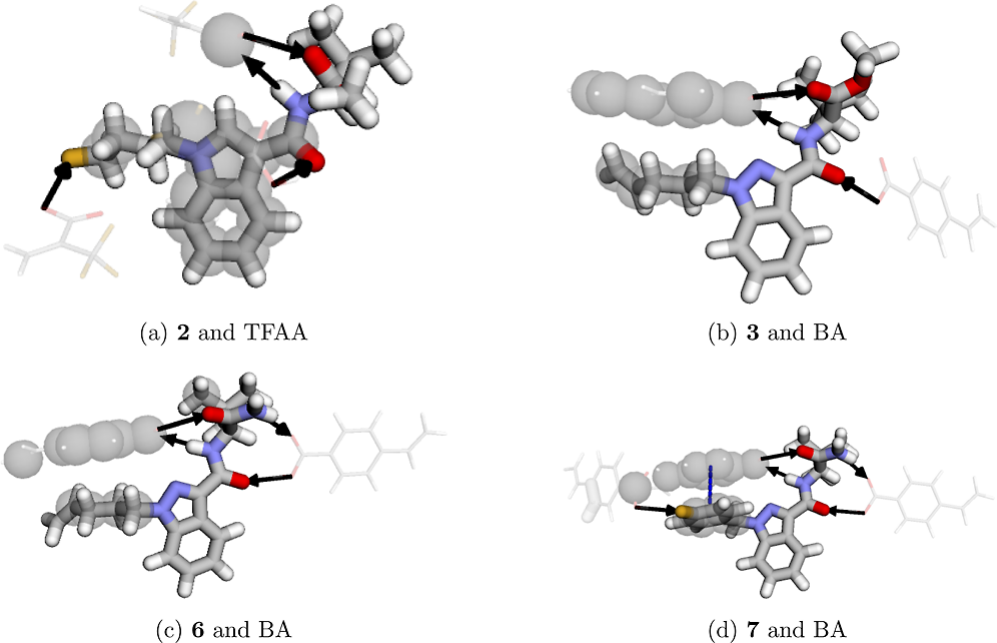
Representatives of each interaction group with their respective strongest interaction energy for a given FM.

Among all the calculated combinations, SC **7** and FM BA provided the strongest interaction of −46.42 kcal/mol. In this case, there is also a π–π stacking interaction between the aromatic ring of BA and the aromatic ring present in the tail moiety (Figure 8d). This interaction occurs when there is an arrangement between the aromatic rings in such a way that they are face-to-face. This new interaction provides great stability to this set. Thus, in a mixture where all SCs are combined, the polymerization process with BA would be preferred for **7**, given its strongest interaction.

### 3.5 Solvent for Washing the MIP

After polymerization, it is necessary to remove the SCs from the surface of the obtained polymer. Another instance where this removal is required is during the application of the MIP. After extracting the intact SC from the seized matrix, it is necessary to remove the SC for analysis, followed by cleaning the MIP for proper reuse.^19^

The most efficient cleaning method involves using a solvent that competes with the MIP for hydrogen bonding interactions with the SC.^39^ Thus, a polar protic solvent is desirable. In this regard, the following solvents were selected: water (H_2_O), methanol (MeOH), ethanol (EtOH), and isopropyl alcohol (*i*-PrOH).

In this case, the use of a continuous solvation model, as applied before for the aprotic solvents, would not be suitable for determining the involved complexation energy because this type of model cannot include hydrogen bonding in its formalism.^40^ Therefore, to construct a more representative system, it is necessary to form a complex between the SC and the solvent, analogous to the treatment applied to the systems of SCs and monomers. Such a complex is shown in Table S7.

Thus, it was observed that, in general, water did not yield satisfactory results (Figure 9 and Table S8): its highly polar nature is incompatible with the structure of the SCs, which, despite being capable of hydrogen bonding, also possess nonpolar regions. On the other hand, isopropanol showed excellent results for all SCs, positioning itself as the most suitable solvent or very close to that position.

**Figure 9:**
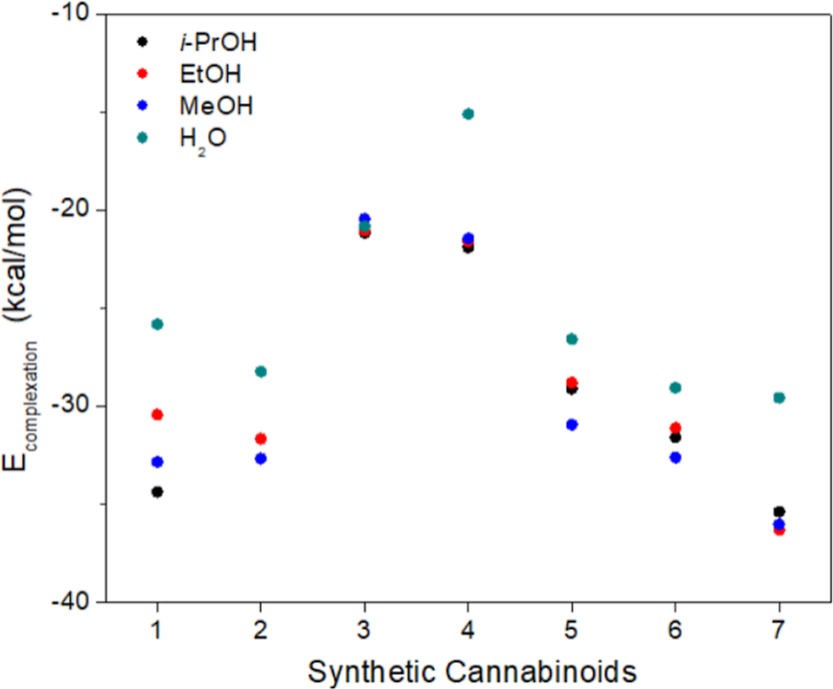
Complexation energy for SCs in protics solvents, water (H_2_O), methanol (MeOH), ethanol (EtOH), and isopropyl alcohol (*i*-PrOH).

## 4 Conclusion

This study provides a comprehensive theoretical framework for designing and optimizing MIPs tailored specifically for new SCs. The analysis identifies the most effective solvents and FMs based on their ability to form stable complexes with SCs, with special emphasis on hydrogen bonding interactions. Geometry optimization calculations were performed using GFN2-xTB, followed by total energy calculations at the ωB97XD/6–31+G­(d,p) level of theory, ensuring accurate structural characterization. Acetone, acetonitrile, and dichloromethane emerged as the most suitable solvents for the extraction and polymerization processes, as evaluated using the SMD solvation model within the DFT framework. At the same time, isopropyl alcohol was identified as the optimal solvent for MIP cleaning, a conclusion supported by explicit solvation calculations, which accounted for strong hydrogen bonding interactions. Among the evaluated FMs, TFAA and BA offered the best stabilization of SCs, with BA showing additional π-stacking interactions that further enhanced its efficacy. This work not only highlights the critical factors influencing MIP performance, such as the structural characteristics of SCs and their interaction patterns with monomers, but also provides practical guidance for the preparation of MIPs, integrating a stepwise computational approach to solvent and monomer selection.

## Supplementary Material



## Notes

All solvation energy values, obtained using the SMD model, electrostatic potential surfaces for compounds **1**–**5** and **7,** all complexation energy values, obtained for SCs and FMs, interactions between all SCs and FMs, as well as explicit solvent molecules, obtained using the Binana software, and all solvation energy values, obtained using explicit molecules solvents (PDF)

## References

[1] Alves V. L., Gonçalves J. L., Aguiar J., Teixeira H. M., Câmara J. S. (2020). The synthetic
cannabinoids phenomenon: from structure to toxicological properties.
A review. Crit. Rev. Toxicol..

[2] Sánchez-Hervás E. (2017). Synthetic
cannabinoids: Characteristics, use and clinical implications. Arch. Psychiatr. Psychother..

[3] De
Luca M. A., Fattore L. (2018). Therapeutic Use of Synthetic Cannabinoids:
Still an Open Issue?. Clin. Ther..

[4] SPSS for Public Security , Novas Substâncias Psicoativas no Estado de São Paulo. https://www.ssp.sp.gov.br/assets/download/Novas%20Substa%CC%82ncias%20Psicoativas_Sa%CC%83o%20Paulo%20Relato%CC%81rio.pdf, 2023; (accessed, 2024 11 13).

[5] de
Oliveira A. S., Antonio A. S., Bhering C. A., Wurzler G. T., de Almeida F. G., Carvalhosa D. R., de Oliveira M. A. M., Neto F. R. d. A., Costa G. V. (2023). Chemical Profile
of Drug Infused Papers Seized in Rio de Janeiro (Brazil) Prisons During
the COVID-19 Lockdown. J. Braz. Chem. Soc..

[6] Köse K., Kehribar D. Y., Uzun L. (2021). Molecularly imprinted
polymers in
toxicology: A literature survey for the last 5 years. Environ. Sci. Pollut. Res..

[7] Mulder H. A., Halquist M. S. (2021). Growing trends in the efficient and
selective extraction
of compounds in complex matrices using molecularly imprinted polymers
and their relevance to toxicological analysis. J. Anal. Toxicol..

[8] Li F., Yue S., Zhao Z., Liu K., Wang P., Zhan S. (2024). Application
of molecularly imprinted polymers in the water environmental field:
A review on the detection and efficient removal of emerging contaminants. Mater. Today Sustain..

[9] Díez-Pascual A.
M. (2023). Perspectives
of Polymers in Forensic Analysis. Macromol.

[10] Gavrila A.-M., Diacon A., Iordache T.-V., Rotariu T., Ionita M., Toader G. (2024). Hazardous Materials from Threats
to Safety: Molecularly
Imprinted Polymers as Versatile Safeguarding Platforms. Polymers.

[11] Cela-Pérez M.
C., Bates F., Jiménez-Morigosa C., Lendoiro E., de Castro A., Cruz A., López-Rivadulla M., López-Vilariño J. M., González-Rodríguez M. V. (2016). Water-compatible
imprinted pills for sensitive determination of cannabinoids in urine
and oral fluid. J. Chromatogr. A.

[12] Sánchez-González J., Odoardi S., Bermejo A. M., Bermejo-Barrera P., Romolo F. S., Moreda-Piñeiro A., Strano-Rossi S. (2018). Development
of a micro-solid-phase extraction molecularly imprinted polymer technique
for synthetic cannabinoids assessment in urine followed by liquid
chromatography–tandem mass spectrometry. J. Chromatogr. A.

[13] Sartore D. M., Vargas Medina D. A., Costa J. L., Lanças F. M., Santos-Neto J. (2020). Automated
microextraction by packed sorbent of cannabinoids
from human urine using a lab-made device packed with molecularly imprinted
polymer. Talanta.

[14] Lendoiro E., De Castro A., Fernández-Vega H., Cela-Pérez M. C., López-Vilariño J. M., González-Rodríguez M. V., Cruz A., López-Rivadulla M. (2014). Molecularly imprinted
polymer for selective determination of Δ9-tetrahydrocannabinol
and 11-nor-Δ9- tetrahydrocannabinol carboxylic acid using LC-MS/MS
in urine and oral fluid. Anal. Bioanal. Chem..

[15] Yeganegi A., Fardindoost S., Tasnim N., Hoorfar M. (2024). Molecularly imprinted
polymers (MIP) combined with Raman spectroscopy for selective detection
of Δ-tetrahydrocannabinol (THC). Talanta.

[16] Akgönüllü S., Battal D., Yalcin M. S., Yavuz H., Denizli A. (2020). Rapid and
sensitive detection of synthetic cannabinoids JWH-018, JWH-073 and
their metabolites using molecularly imprinted polymer-coated QCM nanosensor
in artificial saliva. Microchem. J..

[17] Yang F., Fu D., Li P., Sui X., Xie Y., Chi J., Liu J., Huang B. (2023). Magnetic Molecularly
Imprinted Polymers for the Separation
and Enrichment of Cannabidiol from Hemp Leaf Samples. ACS Omega.

[18] Mohsenzadeh E., Ratautaite V., Brazys E., Ramanavicius S., Zukauskas S., Plausinaitis D., Ramanavicius A. (2024). Design of
molecularly imprinted polymers (MIP) using computational methods:
A review of strategies and approaches. Wiley
Interdiscip. Rev.: Comput. Mol. Sci..

[19] Madikizela L. M., Tavengwa N. T., Tutu H., Chimuka L. (2018). Green aspects in molecular
imprinting technology: From design to environmental applications. Trends Environ. Anal. Chem..

[20] Rajpal S., Mishra P., Mizaikoff B. (2023). Rational in
silico design of molecularly
imprinted polymers: current challenges and future potential. Int. J. Mol. Sci..

[21] Silva W. R., Sote W. O., da Silveira
Petruci J.
F., Batista A. D., Junior M. C. (2022). The use of in silico models for the rationalization
of molecularly imprinted polymer synthesis. Eur. Polym. J..

[22] Fernandes L. S., Homem-De-Mello P., De Lima E. C., Honorio K. M. (2015). Rational design
of molecularly imprinted polymers for recognition of cannabinoids:
A structure-property relationship study. Eur.
Polym. J..

[23] Silva C. F., Menezes L. F., Pereira A. C., Nascimento C. S. (2021). Molecularly
Imprinted Polymer (MIP) for thiamethoxam: A theoretical and experimental
study. J. Mol. Struct..

[24] Maia P. P., Zin L. C., Silva C. F., Nascimento C. S. (2022). Atenolol-imprinted
polymer: a DFT study. J. Mol. Model..

[25] Pereira T. F., da Silva A. T., Borges K. B., Nascimento C. S. (2019). Carvedilol-Imprinted
Polymer: Rational design and selectivity studies. J. Mol. Struct..

[26] Rodrigues T. B., Souza M. P., de Melo
Barbosa L., de Carvalho Ponce J., Júnior L. F. N., Yonamine M., Costa J. L. (2022). Synthetic cannabinoid
receptor agonists profile in infused papers seized in Brazilian prisons. Forensic Toxicol..

[27] Bannwarth C., Ehlert S., Grimme S. (2019). GFN2-xTB—An
accurate and broadly
parametrized self-consistent tight-binding quantum chemical method
with multipole electrostatics and density-dependent dispersion contributions. J. Chem. Theory Comput..

[28] Chai J.-D., Head-Gordon M. (2008). Long-range corrected hybrid density functionals with
damped atom–atom dispersion corrections. Phys. Chem. Chem. Phys..

[29] Hehre W. J., Ditchfield R., Pople J. A. (1972). Self—Consistent Molecular
Orbital Methods. XII. Further Extensions of Gaussian—Type Basis
Sets for Use in Molecular Orbital Studies of Organic Molecules. J. Chem. Phys..

[30] Frisch, M. J. ; Gaussian09. Revision D.01; Gaussian Inc.: Wallingford CT, 2016.

[31] Marenich A.
V., Cramer C. J., Truhlar D. G. (2009). Universal Solvation Model Based on
Solute Electron Density and on a Continuum Model of the Solvent Defined
by the Bulk Dielectric Constant and Atomic Surface Tensions. J. Phys. Chem. B.

[32] Breneman C.
M., Wiberg K. B. (1990). Determining
atom-centered monopoles from molecular
electrostatic potentials. The need for high sampling density in formamide
conformational analysis. J. Comput. Chem..

[33] Jmol Development Team . Jmol: An Open-source Java Viewer for Chemical Structures in 3D, 2020. http://www.jmol.org/ (accessed Sep 27, 2020).

[34] Ribeiro
Dutra F., Custodio R. (2024). Comparative assessment of the direct
and isodesmic methods for pKa calculation of monocarboxylic acids
using density functional theory. Comput. Theor.
Chem..

[35] Soares B. M., Sodré P. T., Aguilar A. M., Gerbelli B. B., Pelin J. N., Argüello K. B., Silva E. R., de Farias M. A., Portugal R. V., Schmuck C. (2021). Structure optimization
of lipopeptide assemblies for aldol reactions in an aqueous medium. Phys. Chem. Chem. Phys..

[36] Andrews R., Jorge R., Christie R., Gallegos A. (2023). From JWH-018 to OXIZIDS:
Structural evolution of synthetic cannabinoids in the European Union
from 2008 to present day. Drug Test. Anal..

[37] Piletska E. V., Guerreiro A. R., Romero-Guerra M., Chianella I., Turner A. P., Piletsky S. A. (2008). Design of molecular imprinted polymers
compatible with aqueous environment. Anal. Chim.
Acta.

[38] Johnson C. D., Ellam G. (1971). Substituent effects
on the basicity of pyridine. Elucidation of the
electronic character of. beta.-substituted vinyl groups. J. Org. Chem..

[39] Bitas D., Samanidou V. (2018). Molecularly Imprinted Polymers as Extracting Media
for the Chromatographic Determination of Antibiotics in Milk. Molecules.

[40] Norjmaa G., Ujaque G., Lledós A. (2022). Beyond continuum
solvent models in
computational homogeneous catalysis. Top. Catal..

