# Supplementary material for: Computational-Assisted Development of Molecularly Imprinted Polymers for Synthetic Cannabinoid Recognition
Source: ACS Omega. 2025 Jul 24;10(30):33220–6. doi: 10.1021/acsomega.5c03148 (PMC12332744; doi:10.1021/acsomega.5c03148)
Supplement: Supplementary file 1 [file ao5c03148_si_001.pdf]

Supporting Information

for

Computational-Assisted Development of Molecularly Imprinted Polymers for Synthetic Cannabinoid Recognition

Leonardo Martins Carneiro,† Karen Rafaela Gonçalves Araújo,‡ Diego Ulysses de Melo,¶ Fernando Heering Bartoloni,† Alexandre Learth Soares,§ Mauricio Yonamine,‡ and Paula Homem-de-Mello*,†

†*Centro de Ciências Naturais e Humanas, Universidade Federal do ABC, Santo André, SP 09210-580, Brazil*

‡*Department of Clinical and Toxicological Analyses, School of Pharmaceutical Sciences, University of São Paulo, SP 05508-000, Brazil*

¶*Department of Organic Chemistry, Institute of Chemistry, Universidade Federal Fluminense, Outeiro São João Batista, Niterói, RJ 24220-900, Brazil*

§*Superintendence of the Technical-Scientifc Police, Institute of Criminalistics, São Paulo, SP 05507-060, Brazil*

E-mail: paula.mello@ufabc.edu.br

List of Contents

	Page
**Table S1.** Values of $G_{sol}(A^-)$ and $G_{sol}(HA)$ for each monomer studied in Hartree, as well as the difference between these values in kcal/mol, calculated by $\Delta G_{sol} = [G_{sol}(HA) - G_{sol}(A^-)] \times 627.5$	S3
**Table S2.** Experimental and theoretical pKa values obtained for the monomers studied in this work. The calculated values at the theoretical level ωB97XD/aug-cc-TZVP/SMD(water) in the presence of two explicit water molecules.	S4
**Table S3.** Solvation energy values, obtained using the SMD model, for SCs and aprotics solvents.	S4
**Table S4.** Molecular structures of compounds 1 to 5 and their electrostatic potential surfaces	S5
**Table S5.** Complexation energy values, obtained for SCs and FMs	S6
**Table S6.** Interactions between all synthetic cannabinoids and functional monomers obtained using the Binana software	S7
**Table S7.** Interactions between all synthetic cannabinoids and explicit solvent molecules obtained using the Binana softwaree	S8
**Table S8.** Solvation energy values, obtained using explicit molecules protic solvents	S9

The pK_a value, the acid dissociation equilibrium constant, can be obtained from Equation 1, where R is the gas constant, T is the temperature, and $G_{sol}(\mathrm{A}^-)$, $G_{sol}(\mathrm{HA})$, and $G_{sol}(\mathrm{H}^+)$ represent the Gibbs energies of the conjugate base (A^-), the acid (HA), and the proton in solution (H^+), respectively.

$$pK_{a_{calc}} = \frac{\Delta G_R}{\ln 10 \times RT} = \frac{G_{\mathrm{sol}}(H^+) - G_{\mathrm{sol}}(HA) + G_{\mathrm{sol}}(A^-)}{\ln 10 \times RT}$$

Computationally, we obtained the values for $G_{sol}(\mathrm{A}^-)$ and $G_{sol}(\mathrm{HA})$ from geometry optimization calculations followed by natural frequency analysis, for the ionic and neutral forms of the acid, respectively. For more accurate results, geometry optimization was performed with the SMD solvent model for water, including the presence of two explicit water molecules, at the theoretical level ωB97XD, the same as used throughout the study, with a large basis set, aug-cc-TZVP, as indicated in the literature,[1] Table S1. The value of $G_{sol}(\mathrm{H}^+)$ is very difficult to obtain computationally; therefore, it is common to parameterize it according to the functional applied. In this study, we will use the 274.7 kcal/mol value obtained by Custodio et al.[1]

Table S1: Values of $G_{sol}(\mathrm{A}^-)$ and $G_{sol}(\mathrm{HA})$ for each monomer studied in Hartree, as well as the difference between these values in kcal/mol, calculated by $\Delta G_{sol} = [G_{sol}(\mathrm{HA})$ - $G_{sol}(\mathrm{A}^-)] \times 627.5$

	G_{sol}(HA) hartree	G_{sol}(A^-) hartree	Δ G_{sol} kcal/mol
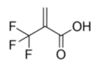 2-(trifluoromethyl)acrylic Acid	-757.115	-756.671	278.53
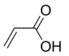 Acrylic Acid	-420.031	-419.583	281.53
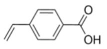 4-vinylbenzoic Acid	-651.012	-650.563	281.82
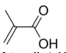 Metacrylic Acid	-459.325	-458.876	281.97

The calculated values were very close to the experimental values, Table S2. In addition to differing by no more than 1 pKa unit, the values maintain the same order of magnitude. This result demonstrates that the acidity of each monomer studied was adequately modeled.

Table S2: Experimental and theoretical pKa values obtained for the monomers studied in this work. The calculated values at the theoretical level ωB97XD/aug-cc-TZVP/SMD(water) in the presence of two explicit water molecules.

	pK_a Exp.	pK_a Calc.	Δ pK_a
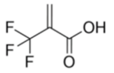 2-(trifluoromethyl)acrylic Acid	2.70 [a]	2.81	0.11
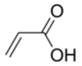 Acrylic Acid	4.20 [a]	5.02	0.82
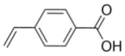 4-vinylbenzoic Acid	4.29 [b]	5.23	0.94
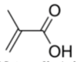 Metacrylic Acid	4.66 [a]	5.33	0.67

[a] From Ref.[2] [b] From Ref.[3]

Table S3: Solvation energy values, obtained using the SMD model, for SCs and aprotic solvents.

Synthetic Cannabinoids	Solvents					
	Ace	ACN	Chl	DCM	EtOEt	DMSO
1	-24,77	-24,28	-21,84	-24,43	-21,34	-20,82
2	-24,1	-23,66	-21,05	-23,65	-20,62	-20,46
3	-19,47	-18,85	-17,7	-19,63	-17,41	-15,37
4	-18,21	-17,52	-16,7	-18,38	-16,53	-14,32
5	-21,51	-21,08	-19,17	-21,33	-18,56	-17,88
6	-20,34	-19,98	-18,42	-20,49	-17,65	-16,41
7	-21,08	-20,75	-19,27	-21,45	-18,46	-16,86

Table S4: Molecular structures of compounds 1 to 5 and their electrostatic potential surfaces.

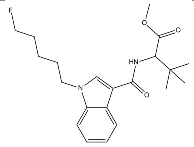 5F-MDMB-Pica (1)	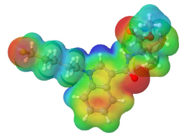
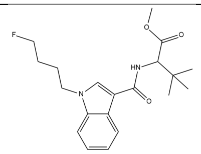 4F-MDMB-Butinaca (2)	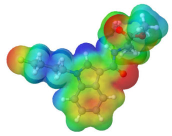
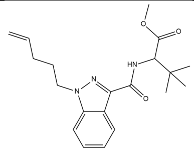 MDMB-4en-Pinaca (3)	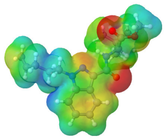
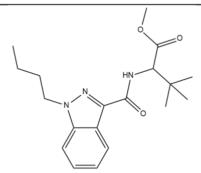 MDMB-Butinaca (4)	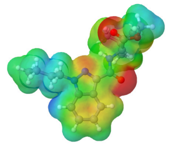
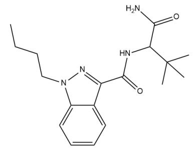 ADB-Butinaca (5)	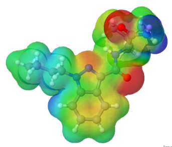
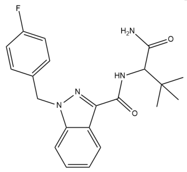 ADB-Fubinata (7)	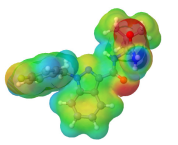

Table S5: Complexation energy values, obtained for SCs and FMs

Synthetic Cannabinoids	Acids			
	**Acrylic**	**Metacrylic**	**2(trifluormethyl) acrylic**	**4-vinylbenzoic**
**1**	-40,81	-37,84	-47,23	-44,91
**2**	-38,22	-41,11	-44,06	-40,29
**3**	-26,04	-27,34	-27	-30,45
**4**	-26,84	-24,6	-27,96	-30,84
**5**	-34,86	-35,79	-37,68	-38,2
**6**	-37,37	-38,23	-39,85	-40,86
**7**	-39,58	-40,18	-41,55	-46,42

Table S6: Interactions between all synthetic cannabinoids and functional monomers obtained using the Binana software.

	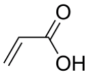 **Acrylic Acid**	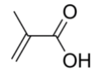 **Metacrylic Acid**	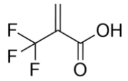 **2-(trifluoromethyl)acrylic Acid**	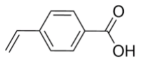 **4-vinylbenzoic Acid**
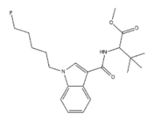 **5F-MDMB-Pica (1)**	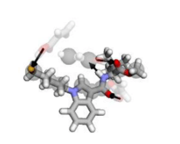	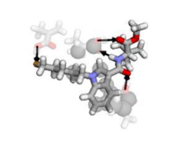	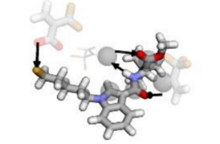	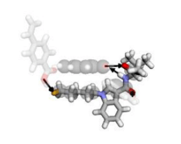
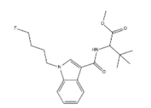 **4F-MDMB-Butinaca (2)**	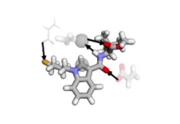	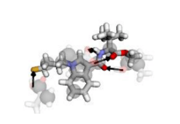	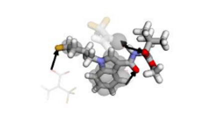	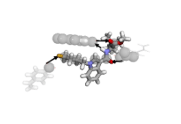
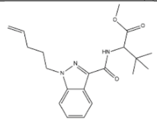 **MDMB-4en-Pinaca (3)**	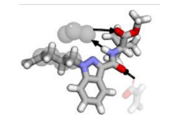	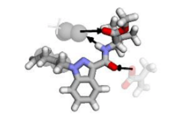	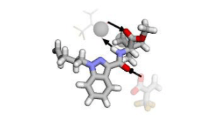	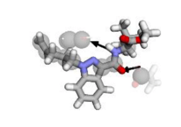
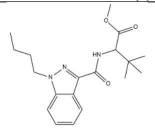 **MDMB-Butinaca (4)**	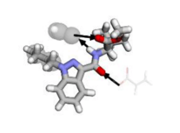	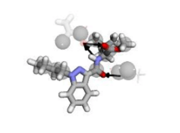	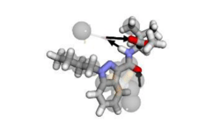	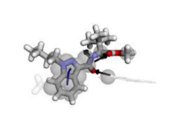
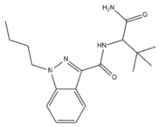 **ADB-Butinaca (5)**	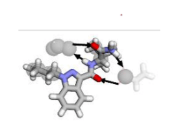	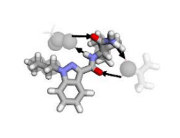	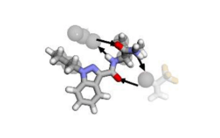	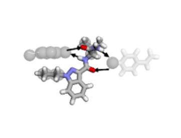
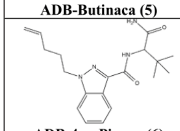 **ADB-4en-Pinaca (6)**	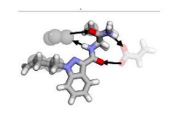	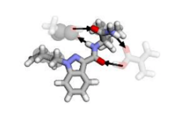	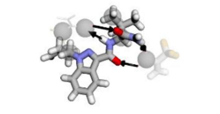	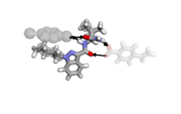
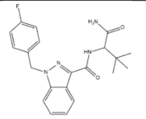 **ADB-Fubinaca (7)**	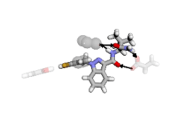	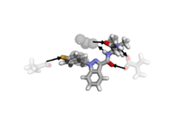	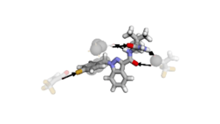	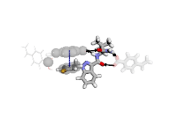

Table S7: Interactions between all synthetic cannabinoids and explicit solvent molecules obtained using the Binana software

	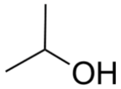 Isopropanol	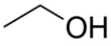 Ethanol	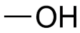 Methanol	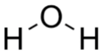 Wataer
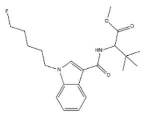 5F-MDMB-Pica (1)	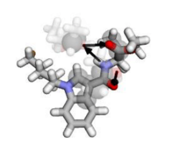	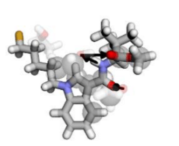	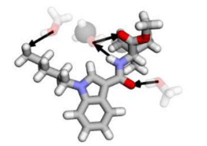	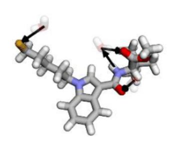
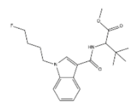 4F-MDMB-Butinaca (2)	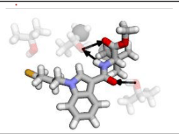	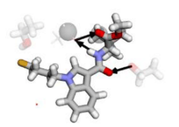	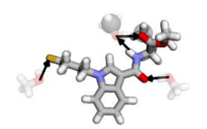	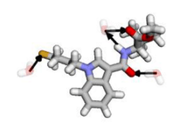
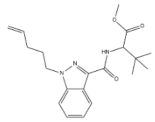 MDMB-4en-Pinaca (3)	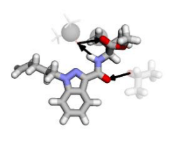	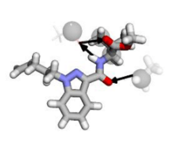	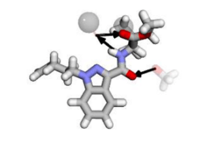	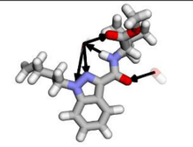
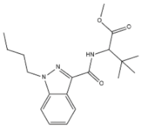 MDMB-Butinaca (4)	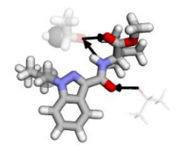	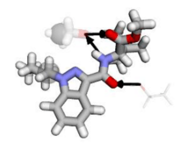	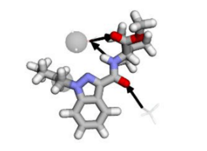	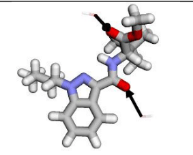
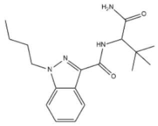 ADB-Butinaca (5)	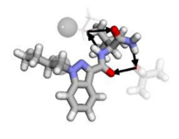	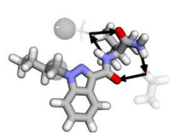	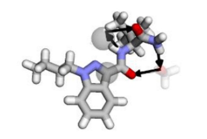	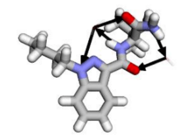
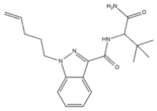 ADB-4en-Pinaca (6)	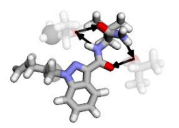	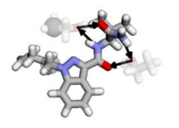	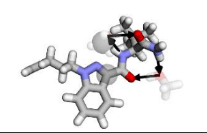	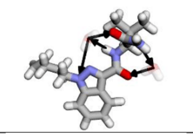
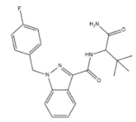 ADB-Fubinaca (7)	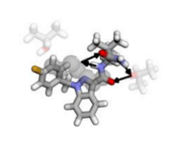	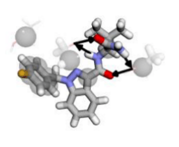	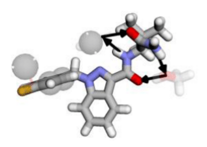	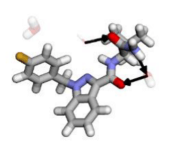

Table S8: Solvation energy values, obtained using explicit molecules protic solvents

Synthetic Cannabinoids	Solvents			
	i-PrOH	EtOH	MeOH	H2O
**1**	-34,37	-30,43	-32,84	-25,82
**2**	-31,67	-31,66	-32,67	-28,24
**3**	-21,15	-21,04	-20,45	-20,83
**4**	-21,89	-21,59	-21,45	-15,1
**5**	-29,11	-28,8	-30,94	-26,59
**6**	-31,58	-31,1	-32,62	-29,06
**7**	-35,38	-36,31	-36,02	-29,57

References

(1) Dutra, F. R.; Custodio, R. Comparative assessment of the direct and isodesmic methods for pKa calculation of monocarboxylic acids using density functional theory. *Computational and Theoretical Chemistry* **2024**, *1237*, 114629.

(2) Piletska, E. V.; Guerreiro, A. R.; Romero-Guerra, M.; Chianella, I.; Turner, A. P.; Piletsky, S. A. Design of molecular imprinted polymers compatible with aqueous environment. *Analytica chimica acta* **2008**, *607*, 54–60.

(3) Johnson, C. D.; Ellam, G. Substituent effects on the basicity of pyridine. Elucidation of the electronic character of. beta.-substituted vinyl groups. *The Journal of Organic Chemistry* **1971**, *36*, 2284–2288.
